# Updating the Northern Tsetse Limit in Burkina Faso (1949–2009): Impact of Global Change

**DOI:** 10.3390/ijerph7041708

**Published:** 2010-04-15

**Authors:** Fabrice Courtin, Jean-Baptiste Rayaissé, Issa Tamboura, Oumar Serdébéogo, Zowindé Koudougou, Philippe Solano, Issa Sidibé

**Affiliations:** 1 Institut de Recherche pour le Développement (IRD), UMR 177 IRD-CIRAD, Centre International de Recherche Développement sur l’Élevage en zone Subhumide (CIRDES), 01 BP 454, Bobo-Dioulasso, Burkina Faso; E-Mail: courtinfabrice@yahoo.fr; 2 Centre International de Recherche Développement sur l’Elevage en zone Subhumide (CIRDES), 01 BP 454, Bobo-Dioulasso, Burkina Faso; E-Mails: jbrayaisse@hotmail.com (J.-B.R.); sambo@fasonet.bf (I.S.); 3 PATTEC-PCZLD, Bobo-Dioulasso, 01 BP 1087, Burkina Faso; E-Mails: issayero_tamboura@yahoo.fr (I.T.); serdebeogo@hotmail.com (O.S.); zowinde@yahoo.com (Z.K.)

**Keywords:** tsetse, northern distribution limit, Burkina Faso, population growth, climate change, control

## Abstract

The northern distribution limit of tsetse flies was updated in Burkina Faso and compared to previous limits to revise the existing map of these vectors of African trypanosomiases dating from several decades ago. From 1949 to 2009, a 25- to 150-km shift has appeared toward the south. Tsetse are now discontinuously distributed in Burkina Faso with a western and an eastern tsetse belt. This range shift can be explained by a combination of decreased rainfall and increased human density. Within a context of international control, this study provides a better understanding of the factors influencing the distribution of tsetse flies.

## 1. Introduction

Tsetse flies (*Diptera: Glossinidae*) are found over much of sub-Saharan Africa, in an area approaching 10 million km^2^. The trypanosomes they transmit, after a cyclical development, affect human welfare, both directly through chronic (due to *Trypanosoma brucei gambiense*) and acute (*T. brucei rhodesiense*) forms of sleeping sickness (SS, or human African trypanosomiasis, HAT), and indirectly through wasting diseases of livestock (animal African trypanosomiases, AATs). AATs also occur outside Africa, where they have been shown to be transmitted mechanically by other biting flies in Asia, South America, and recently in Europe [1].

It has been estimated that the economic benefits accruing to Africa from the eradication of tsetse could reach US$ 4.5 billion per year [2]. HAT is one of the World Health Organization’s (WHO) defined Neglected Tropical Diseases (see http://www.who.int/neglected_diseases/en/); only a small number of (toxic) drugs can cure HAT. There are few prospects of new drugs being developed, and an increasing problem of drug resistance. Accordingly, the only way that trypanosomiases of both humans and livestock can currently be eliminated is through an integrated strategy combining diagnosis and treatment together with vector control (reviewed in [3]). Recently, the African Union (AU) has launched the Pan African Tsetse and Trypanosomiasis Eradication Campaign (PATTEC), a continent-wide initiative that focuses on the progressive elimination in discrete tsetse-infested areas. This vector control program aims to use a variety of methods based on fly species and agro-ecology, including the capacity of local and national tsetse control agencies [4].

Tsetse rely on two major factors for their survival [5]: high local hygrometry and blood from human and mammalian (or reptile) hosts, since both sexes are hematophagous and therefore both can transmit trypanosomes. These tsetse, especially the most dangerous ones of the palpalis group, are intimately related to water (reviewed in [6]). *Glossina palpalis gambiensis* and *G. tachinoides* are riverine tsetse species, which disperse mainly along rivers and in forest vegetation, and only very rarely leave this habitat due to their hygrometric needs [7]. Riverine forests comprise their resting and reproductive sites [8,9]. In savannah areas, their survival is conditioned by the availability of ground water, that in turn governs the persistence of forest vegetation [7,10].

Climate change, population growth and economic development that occurred in West Africa during the 20th century had major consequences on human settlements and the landscape. These changes, in turn, have had an impact on the pathogenic systems of human and animal trypanosomiases, as in other vector-borne diseases [11–13]. Since the last century, within West Africa, the foci of SS have shifted from the savannah areas (where SS no longer prevails) to the forest and mangrove areas, but animal trypanosomiases are still present in the savannah areas [14,15]. There has been a dramatic decrease in tsetse of the *G. morsitans* group (which are usually called savannah tsetse) as a direct result of an increase in human density and its consequences on wildlife, which make up the main blood meal source of this group of tsetse species [10,16]. On the other hand, tsetse species such as *G. palpalis* (which are called riverine or lacustrine) adapt to high human density and are still found in the largest urban centers in West Africa [17].

Despite the importance of such information, in many African tsetse-infested countries the most widely used tsetse distribution maps were established several decades ago [18]. More recently, the FAO-PAAT Information System identified predicted areas of suitability for tsetse flies in Africa, taking into account demographic (human, cattle) and ecological factors (Normalized Difference Vegetation Index, land surface temperature) [19]. Still at the scale of the African continent, a recent study defined landscapes that are more or less favorable to tsetse presence/density, showing the role of vegetation and human activities on tsetse distribution [20]. The use of global geospatial data sets for large- and local-scale African trypanosomiasis management is currently being developed [21,22].

In Burkina Faso, the most recent tsetse distribution map available at the country scale was drawn up by Challier and Laveissière [23]. Taking into account the needs of the national PATTEC program in Burkina Faso (Programme de Création de Zones Libérées Durablement de la Tsé-Tsé, PCZLD), together with recent global changes, the aim of this study – jointly undertaken by PCZLD and CIRDES -was therefore to update the northern tsetse distribution limit in Burkina Faso, to compare the results with previous data recorded more than 50 years ago, and to understand the main causes of the changes of this northern limit. In this paper, we will address the three main tsetse species present in the country: two riverine species belonging to the *G. palpalis* group (*G. palpalis gambiensis* and *G. tachinoides*) and one savannah species belonging to the *G. morsitans* group (*G. morsitans submorsitans*).

## 2. Results and Discussion

### 2.1. Previous Northern Tsetse Limits

**Figure 1.**
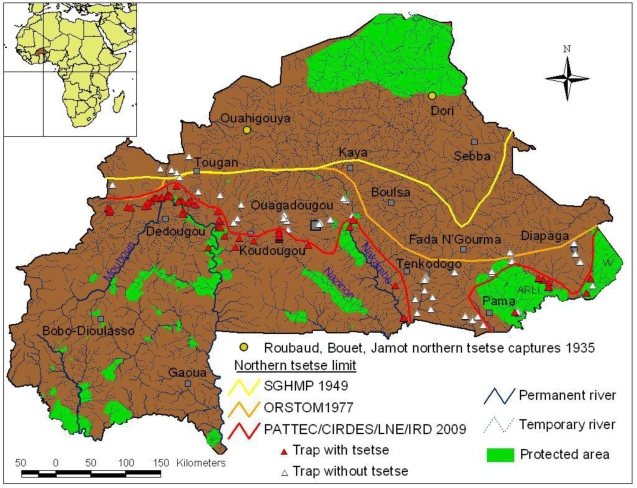
Northern tsetse limit in Burkina Faso from 1906 to 2009.

During the colonial period, Dr. Paul Gouzien (1908) reported the presence of *G. morsitans* and *G. palpalis* in the Dori region [24]. This observation was later confirmed by Roubaud *et al.* [25], who caught tsetse flies (*G. palpalis*) at the latitudes of Ouahigouya (13°30′N) and Dori (14°N) (Figure 1). In 1949, the Service Géographique de l’Afrique Occidentale Française drew up the results of entomological surveys conducted by the Service Général d’Hygiène Mobile et de Prophylaxie (SGHMP,1944–1949), the organization responsible for the control of SS [26]. The northern limit of tsetse flies was formed by *G. tachinoides* and is shown in Figure 1. The limit followed a line starting at the latitude of the town of Tougan (13°N) and then joined Kaya at the same latitude. From Kaya, the limit shifted toward Fada N’Gourma at 12°30′N and then shifted toward Sebba (13°30′N). As illustrated, the shift between the northern-most captures of Roubaud *et al.* [25] (Dori 14°N) and the northern limit of SGHMP in 1949 [26] (Fada N’Gourma, 12°30′N) is roughly 1°30′ to the South (∼145 km).

The map shows the country of Burkina Faso with its main rivers in blue and protected areas (game parks) in green. It also shows the successive tsetse northern distribution limits in 1935, 1949 and 1977, together with results of trapping obtained in the present work, which allows an update and definition of the current tsetse northern limit in Burkina Faso. This clearly illustrates the north-south shift of this northern limit, which is variable according to longitude, and which now results in the present of two distinct tsetse belts in Burkina Faso.

The limit given by Challier and Laveissière in 1977 [23] is also illustrated in Figure 1. In the western part from the Mali–Burkina Faso border to Kaya, the northern tsetse limit did not change between 1949 and 1977 (see Figure 1). However, from Kaya to the east Burkina Faso–Benin border there was a substantial shift toward the south, illustrated by the example of the shift between the latitude of Sebba (13°30′N) in 1949 to Diapaga (12°N) in 1977, equivalent to 1°30′ (∼145 km). As can be seen from 1916 to 1949, and then from 1949 to 1977, the northern tsetse fly limit has shifted north to south but this shift has varied depending on the longitude.

### 2.2. Current Northern Tsetse Limit in Burkina Faso

The updated limits of the most northern points in which tsetse were found present or absent in traps were added to these previously recorded limits, comprising the northern points at which (Figure 1). In the western part of the country (Mouhoun basin), *G. palpalis gambiensis* was caught as well as *G. tachinoides* and *G. morsitans submorsitans*. In the central part of the country (Ouagadougou region), only *G. tachinoides* was caught. In the eastern region of Burkina Faso (Po, Pama, Arly), *G. morsitans submorsitans* was present with *G. tachinoides*, especially in the protected areas (national parks, game reserves), but also in the savannahs. Hence, in the Mouhoun basin it appears that *G. palpalis* occurs mainly upstream (up to the Mouhoun loop north of the Mouhoun River), while *G. tachinoides* were caught downstream. Both species co-occur in the intermediary area east of the Mouhoun river basin, except in the protected areas where *G. morsitans submorsitans* were caught, all the collected flies were only *G. tachinoides*. According to the results of the present surveys, the northwestern tsetse limit starts at the same latitude as the Challier and Laveissière 1977 limit up to the Mouhoun loop, where the limit then proceeds south following the Mouhoun River. Therefore, the limit around Koudougou is now around 12°25′N, and then crosses South Ouagadougou before shifting north again along the Nakambe River (previously known as the Red Volta). At the Nakambe longitude (1°W), the limit shifts right to the south toward Tenkodogo (11°65N) before disappearing at the Togo border (11°N) in this region. This means that here the northern tsetse limit is no longer in Burkina Faso but found in Togo, over a distance of 100 km. This is important when attempting to control tsetse since it means that there are now two separate tsetse belts in Burkina Faso. Tsetse (*G. morsitans submorsitans* and *G. tachinoides*) appear again in Burkina Faso at the Pama longitude (0.80°E) around the Kompienga River and surround the Pama protected areas (Arli). The limit then turns up along the Pendjari River, up to the W game park to the Niger border (12°4′N). Therefore, the most northern point (12°76′N; 03°43′W) at which tsetse have been caught in Burkina Faso is located just north of the Mouhoun loop, in the western part of the country. The most northern points on the other two main rivers are 12°31′N for the Nazinon River and 12°44′N on the Nakambe River.

This update of the northern tsetse limit distribution in Burkina Faso shows that there has been a variable north–south shift of this limit depending on the longitudes. This shift appears to be consistently directed from north to south, never the opposite at this scale. This shift is minimal in the western part of the country, but quite considerable in the east. It can be approximated as a mean of ∼100 km to the south, but is variable according to numerous specific local situations. From 1949 to 2009, the tsetse belt in Burkina Faso lost approximately 70,000 km^2^ (one-quarter of the land surface area in Burkina Faso) due to the combination of a decrease in rainfall and an increase in human density. The largest shift is located in a quadrilateral included within the Tougan-Koudougou-Ouagadougou-Kaya perimeter (the historical focus of SS in Burkina Faso during the 1930s) where tsetse are no longer found today. This is quite impressive when recalling the words of Dr. Gouzien in 1908, who wrote: “Les glossines existent dans la vallée du Niger de Tombouctou à Gaya, dans les marigots de la Boucle, dans la région de Dori, le long de la Sirba et du Yali et aussi en abondance sur les bords du Tchad” [24] - meaning that in a century, tsetse have lost around 500 km of their northern distribution (from the Niger River at Tombouctou to the Mouhoun loop at Dédougou), and are now discontinuously distributed since there are two separate tsetse belts.

It should be noted that, despite rigorous care in the design and implementation of our surveys, it may still be possible that errors have been made in the picture of the tsetse’s northern limit (as is also possible in former surveys). The presence of flies is always much easier to record than their absence (see [27]). However, the aim of this study was to draw a general trend of tsetse distribution in Burkina Faso. Consequently, it cannot be entirely ruled out that isolated populations located north of the proposed limit have been missed, and there may be areas without tsetse below the limit.

### 2.3. Impact of Global Change on Tsetse Distribution

**Figure 2.**
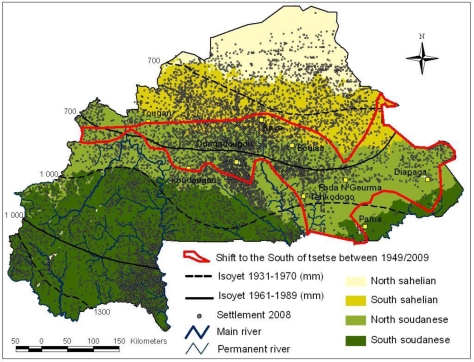
North to south shift of tsetse and rainfall, and settlement changes over time in Burkina Faso.

The West Africa sub-region experienced several drought episodes during the 19th and 20th centuries, which provoked successive famines in the Niger loop [28]. During these droughts, not only rainfall and temperature changed [29], but river flows were also modified [30], which consequently orientated people to the south and along rivers for water, agriculture and livestock tending. In Burkina Faso, three main drought episodes were identified by [31]: 1940–1949, 1970–1979, 1980–1989. Actually, previous entomological data by [23] and [24] were collected during or after these episodes. This shift of tsetse toward the south has therefore been a general process since the beginning of the 20th century that can be partly attributed to these drought episodes. The main result of these drought episodes is the shift of isohyets to the south by 50–200 km depending on the longitude, to which is superimposed the shift of northern tsetse flies limit (Figure 2). The impact of the 1970s drought episodes on tsetse distribution in Burkina Faso has been described in [32].

In the same period, the population of Burkina Faso has undergone a spectacular increase. From 2.8 million inhabitants in 1910 (density 10/km^2^), the population reached 13.9 million in 2005 (51/km^2^) [33,34]. This increase in population density, varying by region, has had a strong impact on vegetation because of the extension of cultivated areas. In Burkina Faso, the Mossi territory located in a quadrilateral within the Ouahigouya-Manga-Tenkodogo-Kaya perimeter surrounding Ouagadougou, is historically known for its high density [35,36] (Figure 2).

This map shows the country of Burkina Faso with its different climatic areas and the changes in isohyets distribution between 1931 and 1989. It illustrates the north-south shift in isohyets distribution, and the variable north-south shift of the northern tsetse limit.

**Figure 3.**
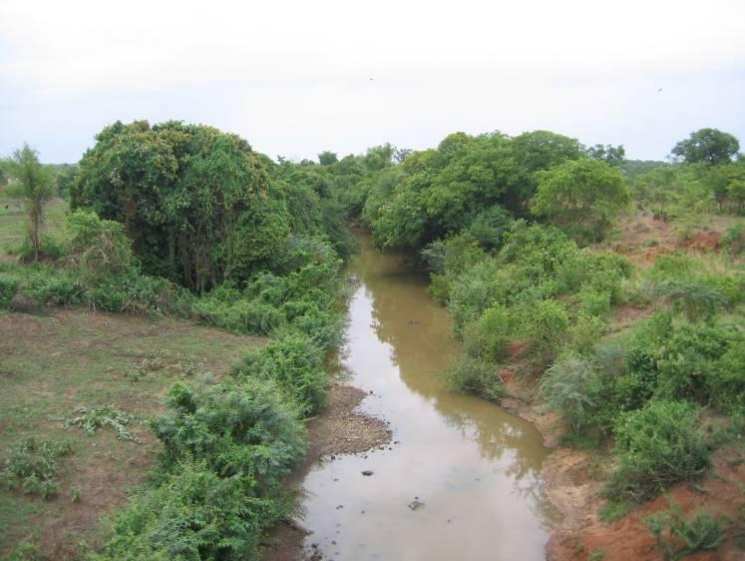
Riverine forest destruction.

In this territory, the population density can reach more than 100/km^2^, and the landscape has been dramatically modified, due to the extension of agriculture, including degradation of gallery forests along rivers (Figure 3), in the same area where the northern tsetse limit has shifted the most toward the south since 1977.

At a first glance (see Figure 1), two factors seem to be slowing down the shift of the distribution limit toward the south: the presence of a permanent hydrographic network and the presence of protected areas. This is particularly the case in the west where the limit has remained relatively stable. Here, this can be explained by two types of factors: (i) macroclimatic, in particular the fact that this area belongs to the South Sudanian zone [37], with the presence of forest galleries, and the presence of the permanent Mouhoun River, which offers (ii) microclimatic conditions (relative hygrometry and host availability) in areas where the gallery has not been destroyed, allowing the survival of riverine tsetse such as *G. palpalis gambiensis* and *G. tachinoides* [10,22]. East of the Mouhoun River, the main rivers are the Nazinon and the Nakambe, and in this area where *G. p. gambiensis* is no longer present, the presence of protected areas and galleries allow the persistence of *G. tachinoides. G. morsitans submorsitans* is only present within the immediate vicinity of protected areas where it can feed on wildlife and cattle, but it has nearly disappeared from areas where the human population has become too high [16,17,38]. The same phenomenon occurred in Senegal on the Somone River (historical sleeping sickness focus), where tsetse disappeared due to intense vegetation degradation along this river after a drought which led people to migrate to this area [39].

Despite this global trend of a decrease in tsetse observed at the scale of Burkina Faso, it should not be forgotten that (i) tsetse do not need to be numerous to be vectors of trypanosomiasis, since small populations can sometimes be much more dangerous to humans than large populations [5], and (ii) opportunistic species of the *G. palpalis* group (*G. palpalis gambiensis* in the savannah, *G. p. palpalis* in the forest) can survive to very high human density levels, since they have been found to adapt to some of the largest towns on the continent, such as Dakar and Abidjan [17]. In addition, tsetse populations have been shown to increasingly transmit trypanosomes when they are submitted to environmental stress [40].

The present study should be extended to other countries of West Africa, in particular those for which a national tsetse control campaign is planned. The existence of models attempting to predict areas of tsetse suitability [21] is helpful; however, for operational control updating tsetse presence in the field is undoubtedly mandatory since substantial differences can occur between the predicted tsetse presence and the actual distribution, as observed in Burkina Faso by [22] and in the present study (data not shown). What will be of key interest to tsetse control planning as a next step could be modeling what has been observed here to predict the future evolution of tsetse and trypanosomiasis, taking into account the climatic scenario [41,42] and projections of human growth [43].

## 3. Experimental Section

Entomological data were recorded from previously reported northern tsetse limits in Burkina Faso. Some were from observations made at the beginning of the last century (Missions Bouët-Roubaud 1906–1916 and Jamot 1933–1935), from which a map was drawn up in 1935 [25]. Later, following other entomological surveys conducted by the SGHMP between 1944 and 1949, another map was prepared in 1949 [26]. Then in 1977, Office de Recherche Scientifique et Technique d’Outre-Mer (ORSTOM) scientists updated these observations [23].

More recently, all entomological data recorded in the last 10 years come from different research projects based at CIRDES Bobo-Dioulasso, the National Livestock Laboratory (LNE) and the Burkina Faso national PATTEC project (PCZLD: Projet de Création de Zones Libérées Durablement de la tsé-tsé). The methodology used by PATTEC-PCZLD is fully described in [44]. Present surveys conducted by CIRDES/LNE also used biconical traps [45] to catch tsetse along the main rivers and their tributaries. Traps were left for three consecutive days, with cages harvested daily. Tsetse were then identified according to species and sex, and counted. Tsetse were noted absent if there were no tsetse caught in the trap over these three consecutive days.

Rainfall data between 1931 and 1970 and between 1961 and 1989 were recorded by the Institut Géographique du Burkina Faso (IGB) and of the Burkina Faso meteorology department. Information on human settlements was recorded from [35] and recent data on settlements were recorded by [36].

## 4. Conclusions

Updating the tsetse northern distribution limit in Burkina Faso and comparing maps made in the beginning of 20th century to the current ones have demonstrated a north-to-south shift of this limit. The maximum shift toward the south since 1949 is around 200 km, and the tsetse belt in Burkina Faso has decreased by around 70,000 km^2^ (one-quarter of the country). Concurrently, the density of several tsetse species has decreased and some of them have almost disappeared, such as *G. morsitans submorsitans*, which only remains present in protected areas (national parks, wildlife areas). The Burkina Faso tsetse belt has now become discontinuous since it is now made up of two belts, one western and one eastern. The first use of these observations is operational since it allows the PATTEC national project to target areas for tsetse control in programs begun on the Mouhoun basin in November 2009.

Two factors seem to have played a major role in this quantitative and qualitative evolution. The severe drought episodes locally modified key factors for tsetse such as vegetation, shade, hygrometry, saturation deficit and temperature [46], and also have led humans to concentrate along rivers. At the same time, the population of Burkina Faso, which has quintupled in the last century, has created high pressure on vegetation and wildlife, and has also strongly modified tsetse habitats.

Taking into account our observations and looking at climate change forecasting for West Africa associated with demographic projections (the population of Burkina Faso is predicted to reach 37.5 million in 2050), identifying areas that will be more or less favorable for tsetse in coming years will be essential, taking into account that riverine species have the potential of adapting to high human density in some cases.
